# Fibroblast gene expression following asthmatic bronchial epithelial cell conditioning correlates with epithelial donor lung function and exacerbation history

**DOI:** 10.1038/s41598-018-34021-6

**Published:** 2018-10-25

**Authors:** Stephen R. Reeves, Kaitlyn A. Barrow, Tessa K. Kolstad, Maria P. White, Lucille M. Rich, Thomas N. Wight, Jason S. Debley

**Affiliations:** 1 Division of Pulmonary and Sleep Medicine, Seattle Children’s Hospital, Seattle, WA USA; 2 Center for Immunity and Immunotherapies, Seattle Children’s Research Institute, Seattle, WA USA; 3 Department of Pediatrics, University of Washington, Seattle, WA USA; 4 Matrix Biology Program, Benaroya Research Institute, Seattle, WA USA

## Abstract

Airway remodeling may contribute to decreased lung function in asthmatic children. Bronchial epithelial cells (BECs) may regulate fibroblast expression of extracellular matrix (ECM) constituents and fibroblast-to-myofibroblast transition (FMT). Our objective was to determine if human lung fibroblast (HLF) expression of collagen I (COL1A1), hyaluronan synthase 2 (HAS2), and the FMT marker alpha-smooth muscle actin (α-SMA) by HLFs conditioned by BECs from asthmatic and healthy children correlate with lung function measures and exacerbation history among BEC donors. BECs from asthmatic (n = 23) and healthy children (n = 15) were differentiated at an air-liquid interface (ALI) and then co-cultured with HLFs for 96 hours. Expression of COL1A1, HAS2, and α-SMA by HLFs was determined by quantitative polymerase chain reaction (qPCR). FMT was quantified by measuring HLF cytoskeletal α-SMA by flow cytometry. Pro-collagen Iα1, hyaluronan (HA), and PGE_2_ were measured in BEC-HLF supernatant. Correlations between lung function measures of BEC donors, and COL1A1, HAS2, and α-SMA gene expression, as well as supernatant concentrations of HA, pro-collagen Iα1, hyaluronan (HA), and PGE_2_ were assessed. We observed that expression of α-SMA and COL1A1 by HLFs co-cultured with asthmatic BECs was negatively correlated with BEC donor lung function. BEC-HLF supernatant concentrations of pro-collagen Iα1 were negatively correlated, and PGE_2_ concentrations positively correlated, with asthmatic BEC donor lung function. Expression of HAS2, but not α-SMA or COL1A1, was greater by HLFs co-cultured with asthmatic BECs from donors with a history of severe exacerbations than by HLFs co-cultured with BECs from donors who lacked a history of severe exacerbations. In conclusion, α-SMA and COL1A1 expression by HLFs co-cultured with BECs from asthmatic children were negatively correlated with lung function measures, supporting our hypothesis that epithelial regulation of HLFs and airway deposition of ECM constituents by HLFs contributes to lung function deficits among asthmatic children. Furthermore, epithelial regulation of airway HAS2 may influence the susceptibility of children with asthma to experience severe exacerbations. Finally, epithelial-derived PGE_2_ is a potential regulator of airway FMT and HLF production of collagen I that should be investigated further in future studies.

## Introduction

Asthma is the most prevalent chronic lung disease of childhood affecting an estimated 14% of the world’s pediatric population^1^. Longitudinal studies in asthmatic children have demonstrated lung function deficits that persist into adulthood^2,3^. One possible mechanism explaining differences in lung function observed between asthmatic and healthy individuals is airway remodeling. Airway remodeling encompasses multiple pathologic changes that have been observed in asthmatic airways^4^. In adult asthma, basement membrane thickening has been well studied and is considered pathognomonic of the disease. Fewer studies of biopsy specimens exist in children; however, both qualitative^5,6^ and quantitative^7,8^ data have demonstrated increased airway basement membrane thickness in children with asthma. Additional studies have shown that basement membrane thickness at infancy does not predict subsequent asthma^9^. Taken together, data from both epidemiologic and pathologic studies support the premise that airway remodeling in asthmatic individuals is not present early in life, but evolves during childhood and persists into adulthood.

There has been increasing focus on the role of the airway epithelium as a driver of asthma pathogenesis given that bronchial epithelial cells (BECs) are the initial point of contact between the environment and the host^10^. Prior work from our laboratory has demonstrated increased expression of pro-remodeling signaling mediators by primary BECs obtained from asthmatic children in well-differentiated air-liquid interface (ALI) cultures^11^. Further studies have demonstrated that healthy human lung fibroblasts (HLFs) co-cultured with differentiated BECs display greater production of extracellular matrix (ECM) components including type I and III collagen, hyaluronan (HA), and fibronectin when co-cultured with primary BECs obtained from asthmatic donors^12^. Separate studies have also confirmed increased expression of alpha smooth muscle actin (α-SMA) and tropomyosin-I from HLFs co-cultured with asthmatic BECs compared to healthy BECs indicative of a greater fibroblast to myofibroblast transition (FMT)^13^.

To investigate potential associations between *ex vivo* BEC regulation of HLFs, and the lung function and exacerbation history of asthmatic BEC donors, we utilized our primary differentiated BEC/HLF co-culture model and medical history and spirometry data from healthy and asthmatic children who donated BECs. We tested the hypothesis that lung function and/or exacerbation history of BEC donors would be associated with the expression of genes related to airway remodeling by HLFs conditioned by BECs from children with asthma. Specifically, we hypothesized that expression of genes related to FMT (α-SMA) and ECM production [collagen I (COL1A1) and hyaluronan synthase 2 (HAS2)] by HLFs co-cultured with BECs would be correlated with spirometry data obtained from BEC donors, and/or associated with a history of severe exacerbations among asthmatic BEC donors. Furthermore, we assessed correlations between prostaglandin E_2_ (PGE_2_) in BEC-HLF supernatant and lung function measures among asthmatic BEC donors given the potential role that this epithelial derived soluble mediator may play in regulating airway stromal cells. Some of the results of these studies have been previously reported in abstract form^14^.

## Results

**Figure 1:**
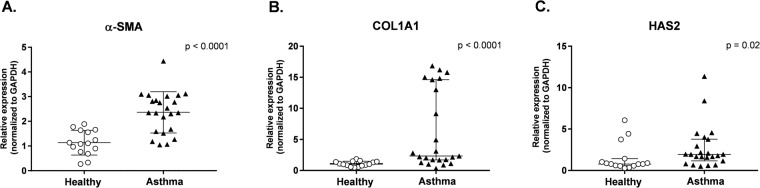
HLF gene expression following co-culture with healthy or asthmatic BECs. Expression of α-SMA (**A**), COL1A1 (**B**), and HAS2 (**C**) by HLFs co-cultured with healthy BECs (open circles, n = 15) or asthmatic BECs (closed triangles, n = 23) presented as scattered plots with lines and error bars representing mean and standard deviation if data was normally distributed. (**A**) Or median with interquartile range if data was not normally distributed. (**B**,**C**) To compare expression of genes between healthy and asthmatic HLF-BEC co-cultures, unpaired t tests were used for normally distributed data, and Mann-Whitney test was used for non-normally distributed data.

Bronchial brushings were obtained from both healthy (n = 15) and asthmatic donors (n = 23) to generate ALI cultures used in BEC/HLF co-cultures. Clinical characteristics for each group are shown in Table 1. Healthy and asthmatic donors were similar in age (11.5 ± 3.3 yrs vs. 12.2 ± 3.0 yrs, respectively) and gender (57% vs. 47% male). The majority of asthmatic subjects displayed atopic features including eczema (48%), allergic rhinitis (83%), and/or positivity to an aeroallergen by radioallergosorbent testing (RAST) IgE testing (78%), and 57% reported using maintenance inhaled corticosteroids at the time of enrollment. Asthmatic subjects had significantly higher levels of fraction of exhaled nitric oxide (FE_NO_) compared to healthy subjects (28.1 ppb in asthmatics vs. 10.5 ppb in healthy controls, *P* = 0.03). Only 5 asthmatic subjects had a bronchodilator response during pulmonary function testing (defined as ≥12% increase in forced expiratory volume in 1 second (FEV_1_)). Asthmatic donors had elevated IgE levels compared to the healthy subjects (mean 273.6 IU/mL vs. 15.3 IU/mL; *P* < 0.001). Forced expiratory volumes and flows were significantly lower in the asthmatic group and consistent with airway obstruction. Expression of α-SMA, COL1A1, and HAS2 by HLFs co-cultured with BECs from healthy (n = 15) and asthmatic (n = 23) children was measured by quantitative polymerase chain reaction (qPCR) and normalized to glyceraldehyde 3-phosphate dehydrogenase (GAPDH). In BEC-HLF co-cultures, α-SMA, COL1A1, and HAS2 expression were all significantly greater by HLFs co-cultured with asthmatic as compared to healthy BECs (Fig. 1). Among HLFs cultured with asthmatic BECs the distribution patterns of expression of these genes differed, with α-SMA expression (Fig. 1A) following a normal distribution, whereas COL1A1 (Fig. 1B) and HAS2 (Fig. 1C) expression was not normally distributed and COL1A1 expression exhibited a bimodal distribution pattern.

**Table 1:** Subject Characteristics.

	Asthmatic Subjects	Healthy Subjects	*P* Value
	*n* = 23	*n* = 15	
Age yrs (mean ± SD)	12.2 (3.0)	11.5 (3.3)	0.5
Sex (% male)	57%	47%	0.6
Currently using inhaled steroids (%)	13 (57%)		
History of Allergic Rhinitis, *n*; (%)	19 (83%)		
History of Eczema, *n*; (%)	11 (48%)		
Positive RAST, *n*; (%)	18 (78%)		
IgE IU/mL (median ± SD)	273.6 (421.2)	15.3 (11.3)	**<0.001**
FVC % predicted (mean ± SD)	100.3 (12.9)	104.7 (12.9)	**0.2**
FEV_1_% predicted (mean ± SD)	87.7 (13.0)	103.9 (11.9)	**0.001**
FEV_1_/FVC Ratio (mean ± SD)	0.79 (0.06)	0.90 (0.1)	**<0.001**
FEF_25–75_% predicted (mean ± SD)	68.5 (24.6)	98.0 (20.8)	**<0.001**
FE_NO_ ppb (mean ± SD)	28.1 (35.7)	10.5 (6.2)	**0.03**

RAST, Radioallergosorbent testing; FVC, Forced vital capacity; FEV_1_, Forced expiratory volume in one second; FEF_25–75_, Forced expiratory flow between 25% and 75% of expiration.

**Figure 2:**
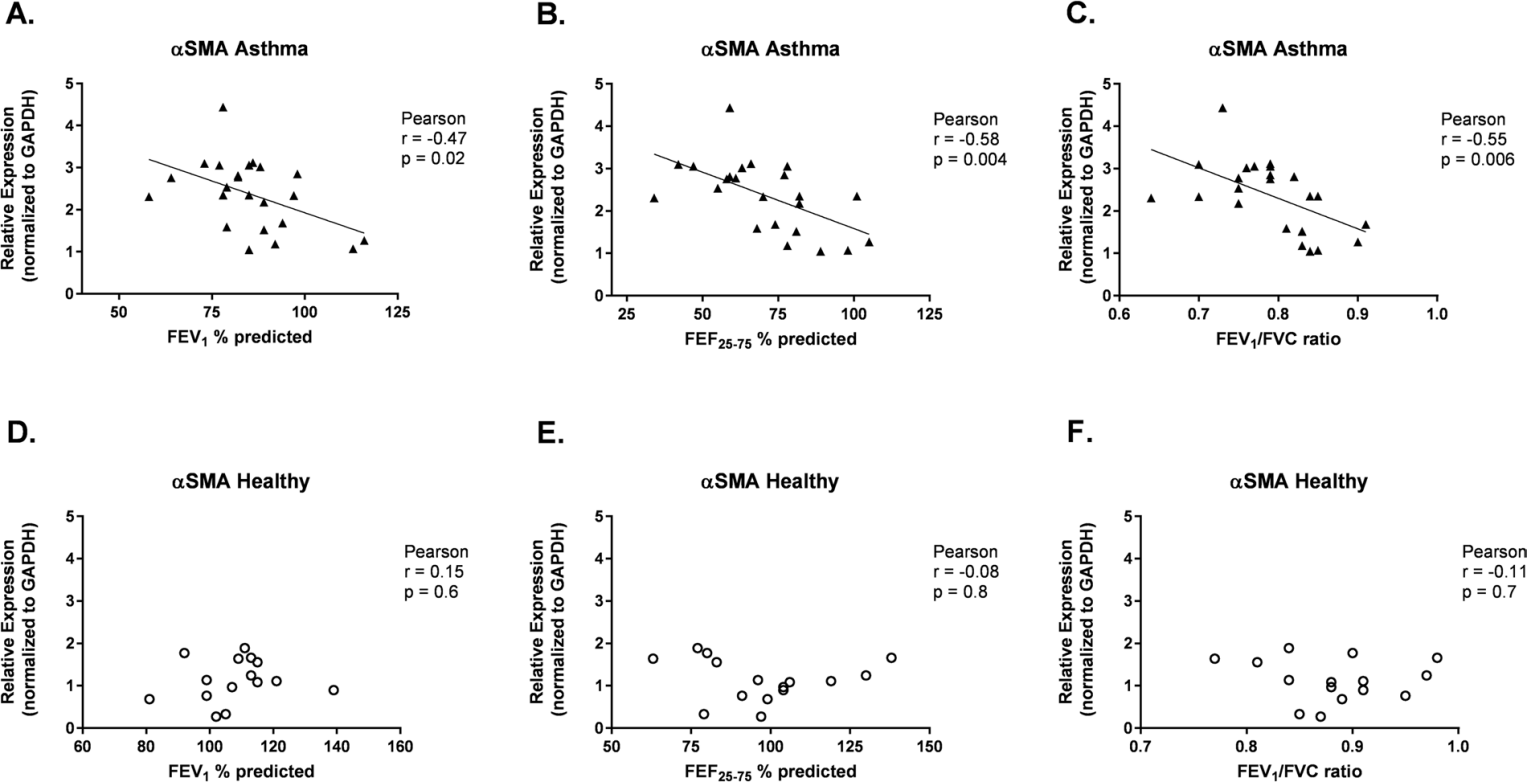
Correlation between HLF expression of α-SMA following co-culture with healthy or asthmatic BECs and lung function measures of BEC donors. Correlations between HLF expression of α-SMA following co-culture with asthmatic BECs and the lung function measures FEV_1_% predicted (**A**), FEF_25–75%_ predicted (**B**), and FEV_1_/FVC ratio (**C**) of asthmatic BEC donors were calculated using the Pearson correlation coefficient. Correlations between HLF expression of α-SMA following co-culture with healthy BECs and the lung function measures FEV_1_% predicted (**D**), FEF_25–75%_ predicted (**E**), and FEV_1_/FVC ratio (**F**) of healthy BEC donors were calculated using the Pearson correlation coefficient.

**Figure 3:**
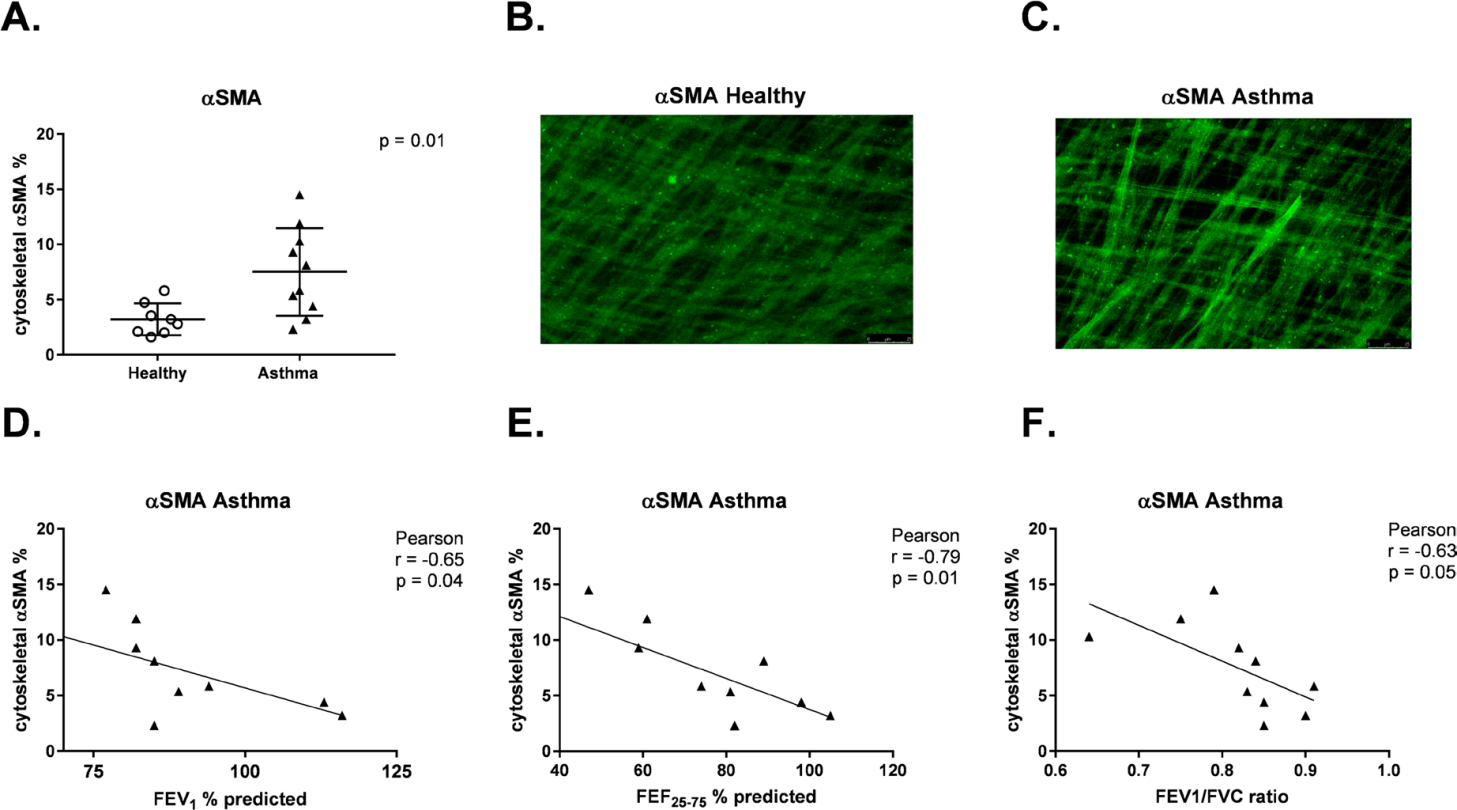
Correlation between percentage of HLFs expressing cytoskeletal α-SMA following co-culture with healthy or asthmatic BECs and lung function measures of BEC donors. Percentage (%) of HLFs expressing cytosketal α-SMA quantified by flow cytometry following co-culture with healthy (n = 8, open circles) and asthmatic (n = 10, open triangles) BECs. (**A**) Representative immunohistochemistry (IHC) image of cytoskeletal α-SMA staining of HLFs co-cutured with healthy BECs. (**B**) Representative IHC image of cytoskeletal α-SMA staining of HLFs co-cutured with asthmatic BECs. (**C**) Correlations between % HLFs expressing cytosketal α-SMA quantified by flow cytometry following co-culture with asthmatic BECs and the lung function measures FEV_1_% predicted (**D**), FEF_25–75%_ predicted (**E**), and FEV_1_/FVC ratio. (**F**) of asthmatic BEC donors were calculated using the Pearson correlation coefficient.

Normalized HLF gene expression for COL1A1, HAS2, and α-SMA was plotted against measures of lung function for each individual BEC donor in the study. Associations between lung function measures by BEC donors and HLF expression of α-SMA, COL1A1, and HAS2 were assessed separately among asthmatic and healthy BEC donors. Among asthmatic BEC donors, expression of α-SMA by HLFs co-cultured with BECs was negatively correlated with FEV_1_ (r = −0.47, *P* = 0.02, Fig. 2A), forced expiratory flow between 25% and 75% of forced vital capacity (FEF_25–75_) (r = −0.58, *P* = 0.004, Fig. 2B), and FEV_1_/forced vital capacity (FVC) ratio (r = −0.55, *P* = 0.006, Fig. 2C). In a subset of healthy (n = 8) and asthmatic (n = 10) BEC-HLF co-cultures, FMT was quantified by measuring HLF cytoskeletal α-SMA by flow cytometry. The percentage of HLFs positive for cytoskeletal α-SMA was significantly greater among asthmatic as compared to healthy co-cultures, and there was a significant negative correlation between the percentage of HLFs positive for cytoskeletal α-SMA among HLFs co-cultured with asthmatic BECs and BEC donor FEV1 (r = −0.65, P = 0.04, Fig. 3D), FEF25–75 (r = −0.79, P = 0.01, Fig. 3E) and FEV1/FVC (r = −0.63, P = 0.05, Fig. 3F).

**Figure 4:**
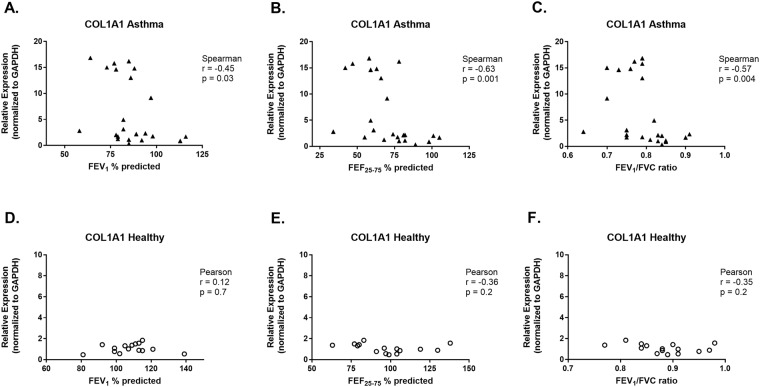
Correlation between HLF expression of COL1A1 following co-culture with healthy or asthmatic BECs and lung function measures of BEC donors. Correlations between HLF expression of COL1A1 following co-culture with asthmatic BECs and the lung function measures FEV_1_% predicted (**A**), FEF_25–75%_ predicted (**B**), and FEV_1_/FVC ratio (**C**) of asthmatic BEC donors were calculated using the Spearman correlation coefficient. Correlations between HLF expression of COL1A1 following co-culture with healthy BECs and the lung function measures FEV_1_% predicted (**D**), FEF_25–75%_ predicted (**E**), and FEV_1_/FVC ratio (**F**) of healthy BEC donors were calculated using the Pearson correlation coefficient.

**Figure 5:**
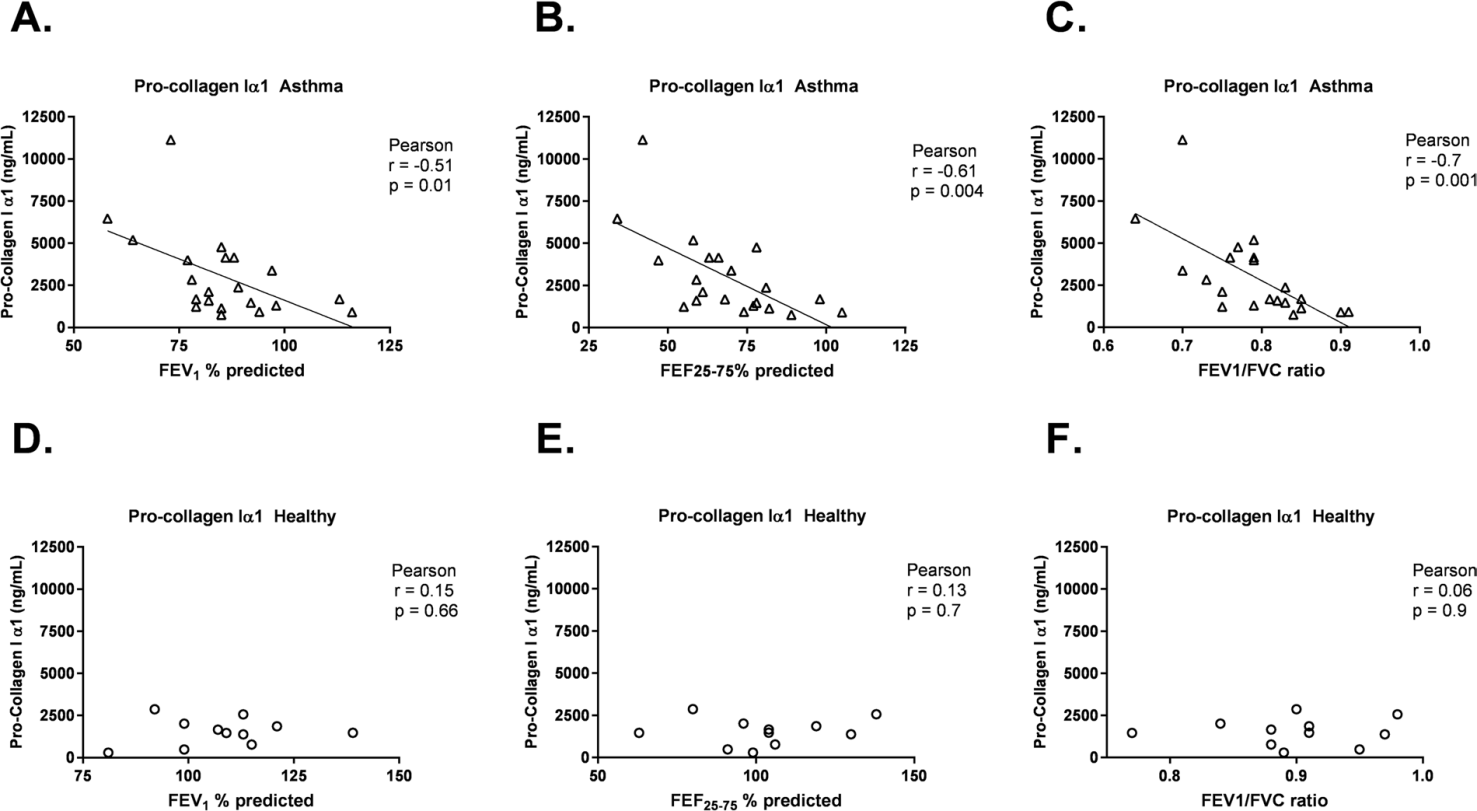
Correlation between Pro-collagen I α1 concentration in BEC-HLF supernatant following HLF co-culture with healthy or asthmatic BECs, and lung function measures of BEC donors. Correlations between the concentration of pro-collagen I α1 in BEC-HLF supernatant following HLF co-culture with asthmatic BECs (n = 21, open triangles) and the lung function measures FEV_1_% predicted (**A**), FEF_25–75%_ predicted (**B**), and FEV_1_/FVC ratio (**C**) of asthmatic BEC donors were calculated using the Pearson correlation coefficient. Correlations between the concentration of pro-collagen I α1 in BEC-HLF supernatant following HLF co-culture with healthy BECs (n = 11, open circles) and the lung function measures FEV_1_% predicted (**D**), FEF_25–75%_ predicted (**E**), and FEV_1_/FVC ratio (**F**) of healthy BEC donors were calculated using the Pearson correlation coefficient.

**Figure 6:**
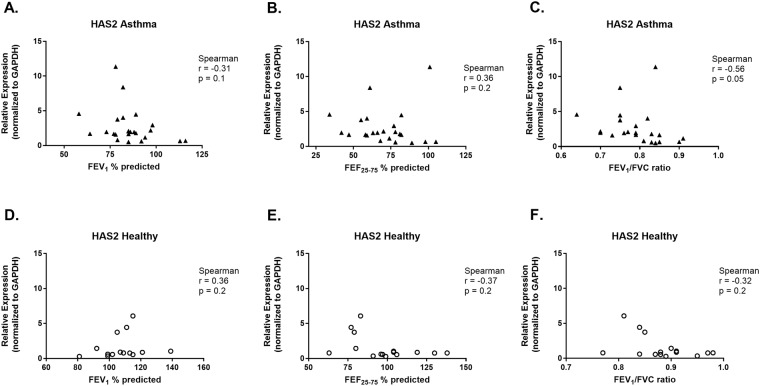
Correlation between HLF expression of HAS2 following co-culture with healthy or asthmatic BECs and lung function measures of BEC donors. Correlations between HLF expression of HAS2 following co-culture with asthmatic BECs and the lung function measures FEV_1_% predicted (**A**), FEF_25–75%_ predicted (**B**), and FEV_1_/FVC ratio (**C**) of asthmatic BEC donors were calculated using the Spearman correlation coefficient. Correlations between HLF expression of HAS2 following co-culture with healthy BECs and the lung function measures FEV_1_% predicted (**D**), FEF_25–75%_ predicted (**E**), and FEV_1_/FVC ratio (**F**) of healthy BEC donors were calculated using the Spearman correlation coefficient.

Expression of COL1A1 by HLFs co-cultured with asthmatic BECs was negatively correlated with FEV1 (r = −0.45, *P* = 0.03, Fig. 4A), FEF_25–75_ (r = −0.63, *P* = 0.001, Fig. 4B) and FEV_1_/FVC (r = −0.57, *P* = 0.004, Fig. 4C). The concentration of pro-collagen I α1 in BEC-HLF supernatant following HLF co-culture with BECs was also negatively correlated with FEV1 (r = −0.51, *P* = 0.01, Fig. 5A), FEF_25–75_ (r = −0.61, *P* = 0.004, Fig. 5B) and FEV_1_/FVC (r = −0.7, *P* = 0.001, Fig. 5C) among asthmatic BEC-HLF co-cultures, whereas supernatant pro-collagen I α1 concentrations were not correlated with healthy BEC donor lung function. There was no correlation between FEV_1_ or FEF_25–75_ of asthmatic BEC donors and HLF expression of HAS2 (FEV_1_: r = −0.31, *P* = 0.1, Fig. 6A; FEF_25–75_: r = 0.36, *P* = 0.2, Fig. 6B), but there was a borderline significant negative correlation between FEV_1_/FVC and HLF expression of HAS2 (FEV_1_/FVC: r = −0.56, *P* = 0.05, Fig. 6C). Among healthy children, there were no significant correlations observed between BEC donor lung function measures and HLF expression of α-SMA, COL1A1, or HAS2.

**Figure 7:**
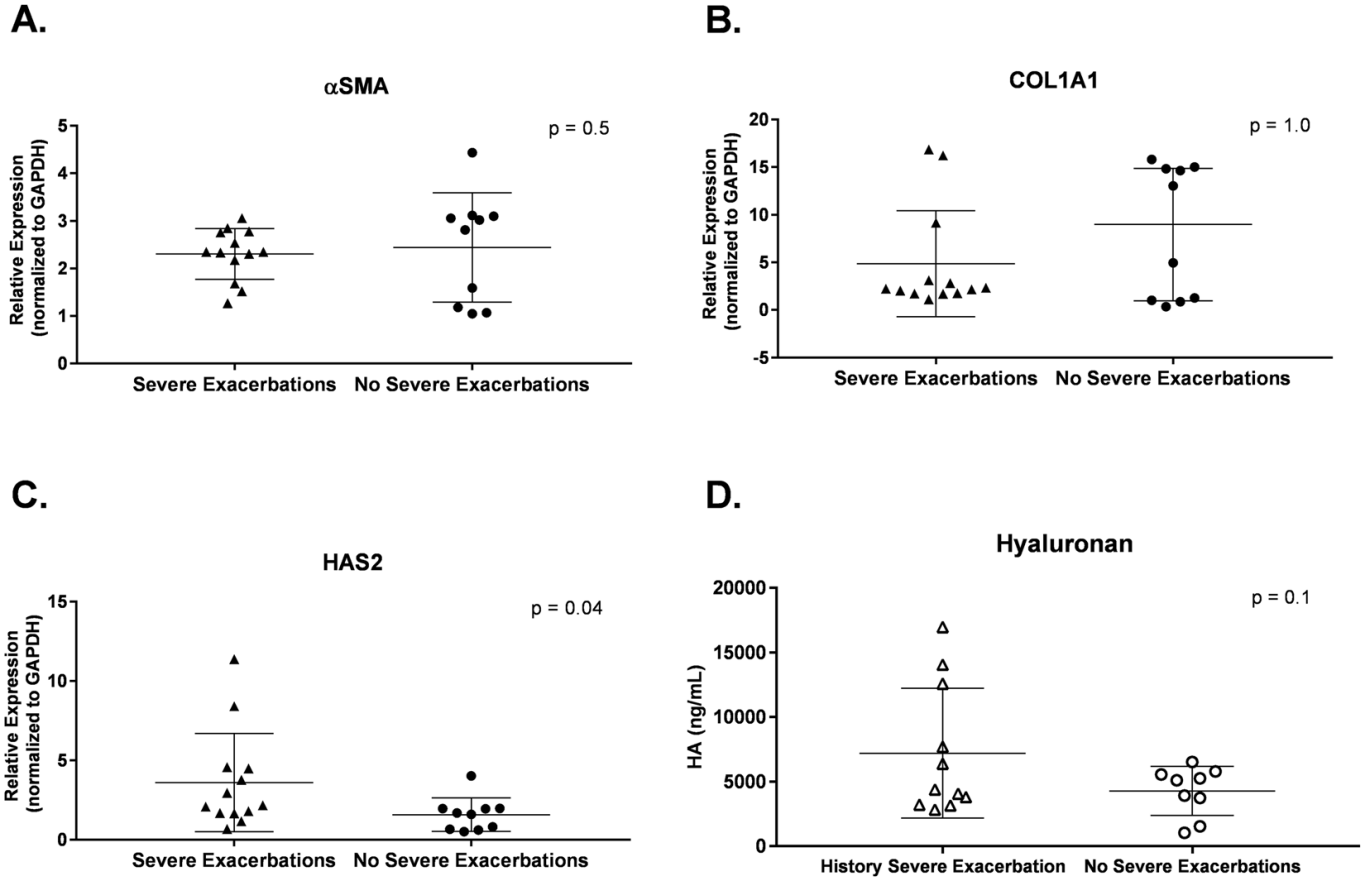
HLF expression of α-SMA, COL1A1, and HAS2, and supernatant concentration of HA, following co-culture with asthmatic BECs among donors with and without history of severe exacerbations. α-SMA expression by HLFs following co-culture with asthmatic BECs among BEC donors with and without a history of severe exacerbations. (**A**) COL1A1 expression by HLFs following co-culture with asthmatic BECs among BEC donors with and without a history of severe exacerbations. (**B**) HAS2 expression by HLFs following co-culture with asthmatic BECs among BEC donors with and without a history of severe exacerbations. (**C**) Hyaluronan (HA) concentration in BEC-HLF supernatant following HLF co-culture with asthmatic BECs (subset, n = 20 co-cultures) among BEC donors with and without a history of severe exacerbations. (**C**) The unpaired t test was used to compare normal distributions (A, α-SMA, HA), and the Mann-Whitney test was used to compare non-normal distributions (B, COL1A1; C, HAS2).

We assessed associations between the expression of α-SMA, COL1A1, and HAS2 by HLFs cultured with asthmatic BECs and history of severe asthma exacerbations among BEC donors, defined as an acute asthma exacerbation requiring treatment with systemic corticosteroids in the emergency department or hospital. Expression of α-SMA and COL1A1 by HLFs cultured with BECs from children with asthma and a history of severe exacerbations was not significantly different than expression by HLFs cultured with BECs from asthmatic children without a history of severe exacerbations (Fig. 7A,B). HAS2 expression was significantly greater in HLFs cultured with BECs from asthmatic donors with a history of severe exacerbations (Fig. 7C, *P* = 0.04), however, concentrations of hyaluronan (HA) in BEC-HLF co-culture supernatant were not significantly different between asthmatic BEC donors with and without a history of severe exacerbations (Fig. 7D, *P* = 0.1).

**Figure 8:**
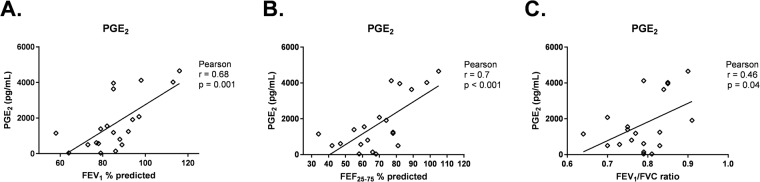
Correlation between PGE_2_ concentration in BEC-HLF supernatant following HLF co-culture with asthmatic BECs, and lung function measures of BEC donors. Correlations between the concentration of PGE_2_ in BEC-HLF supernatant following HLF co-culture with asthmatic BECs (n = 21, open diamonds) and the lung function measures FEV_1_% predicted (**A**), FEF_25–75%_ predicted (**B**), and FEV_1_/FVC ratio (**C**) of asthmatic BEC donors were calculated using the Pearson correlation coefficient.

Finally, we measured concentrations of PGE_2_ in supernatant of asthmatic BEC-HLF supernatant (n = 21) following HLF co-culture with BECs. PGE_2_ concentrations were positively correlated with FEV1 (r = 0.68, *P* = 0.001, Fig. 8A), FEF_25–75_ (r = 0.7, *P* < 0.001, Fig. 8B) and FEV_1_/FVC (r = 0.46, *P* = 0.04, Fig. 8C) among asthmatic BEC donors.

## Discussion

In this study, we have demonstrated that expression of the myofibroblast marker α-SMA and COL1A1 by HLFs co-cultured with primary BECs from asthmatic children, as well as concentrations of soluble collagen I in supernatant from asthmatic BEC-HLF co-cultures, are inversely correlated with objective measures of lung function among asthmatic BEC donors. Furthermore, we observed that among asthmatic BEC donors, HAS2 expression by HLFs co-cultured with BECs was greater when HLFs were co-cultured with BECs from asthmatic donors with a history of severe exacerbation, suggesting that airway HAS2 expression modifies the risk of exacerbation. Finally, consistent with prior observations from our model, expression of α-SMA, COL1A1, and HAS2 was greater by HLFs co-cultured with BECs from asthmatic as compared to healthy donors^12,13^. These findings are novel in that they for the first time provide *ex vivo* human cell culture data obtained using cells from carefully characterized pediatric subjects that further support the paradigm that the airway epithelium influences airway remodeling, and perhaps exacerbation risk, via regulation of mesenchymal cells such as HLFs. A major strength of this study is that our BEC/HLF co-culture model system utilizes primary BECs obtained from clinically-phenotyped children. This model allows for direct correlation of biological outcome measures in our model system with clinical data obtained from study participants.

While multiple cross-sectional and longitudinal studies of lung function in adults exist, fewer studies have examined the longitudinal changes in lung function seen in asthmatic children. Data from the Tucson Children’s Respiratory Study suggest that pulmonary function in children who develop persistent symptoms of asthma is normal at birth, with subsequent evidence of airflow limitation manifesting as early as 6 years^2^. Noteworthy, persistent wheezing was associated with several risk factors for atopy that were not present in the other groups^2^. Separately, the Melbourne Asthma Study enrolled nearly 500 children beginning at the age 7 and has followed the cohort for >50 years^15^. An important observation from the Melbourne cohort is that by age 7 years children who had persistent asthma also had significantly lower lung function compared to healthy subjects. Throughout the 50-year follow-up, lung function measures continued to be lower in the severe asthma group as compared to subjects without asthma, but did not worsen relative to the other groups beyond adolescence. In contrast, other studies have demonstrated lung function decline in adult asthma populations over time^16,17^. Data from the Severe Asthma Research Program (SARP) demonstrated an absolute decline in post-bronchodilator FEV_1_ (% predicted) in longitudinal studies of individuals with severe asthma, which correlated with airway thickness observed on CT scans of the lung^17^. No differences were observed in pre-bronchodilator FEV_1_ (% predicted) in the SARP study.

Accumulating data have provided further insight into the factors contributing to basement membrane thickening in asthmatic airway remodeling. Once thought to be the product of chronic airway inflammation, more recent studies suggest that remodeling may occur in parallel with inflammatory changes or may be independent of airway inflammation^18^. Additional studies have provided insight into mechanisms of disordered wound repair also contributing to remodeling, which may be regulated by the epithelium^10^. From a lung mechanics standpoint, airway remodeling is a biologically plausible explanation for the reduced lung function seen in asthma^19^. While the concept of airway remodeling refers in general to a complex process that includes several pathological features, changes in the basement membrane have been the most extensively described findings in asthmatic airways^20^. Biopsy studies have provided insight into the role of basement membrane thickening and decreased lung function. In adults, evidence of airflow limitation correlated with increased airway wall thickness related to thickening of both the epithelial and basement membrane layers^21^. These findings are consistent with several prior reports of increased deposition of ECM constituents in the subepithelial layer in adults with asthma^22–24^. Payne and colleagues compared basement membrane thickness in airway biopsies obtained from children with asthma with samples obtained from adults with asthma. In that study, the authors demonstrated a similar degree of thickening in samples obtained from asthmatic children compared to adults regardless of asthma severity^7^. These data suggest that establishment of the thickened basement membrane seen in asthma occurs during childhood and persists into adulthood.

Previous work has demonstrated that fibroblasts are the predominant source of many ECM constituents. This is particularly true for HLFs that have undergone FMT^25,26^. Myofibroblasts are a primary source of types I and III collagen in fibrotic lesions and represent a contractile phenotype that may directly participate in scar formation and contraction in the asthmatic airway^27–30^. Other important ECM constituents such as HA are also primarily secreted by fibroblasts and may play important roles in airway remodeling. HA is a major component of the ECM that has been shown in animal models to be involved in inflammatory cell recruitment and retention during wound repair, is directly influenced by transforming growth factor beta (TGFβ) signaling^31^, and its clearance is essential for resolution of local inflammation during acute injury^32,33^. Our observations of greater HLF expression of the HA synthetic enzyme HAS2 when cultured with asthmatic BECs from donors with a history of severe exacerbations is particularly interesting within the context of these prior findings relating HA to inflammatory cell recruitment and retention^31-33^. Furthermore, HA in bronchoalveolar lavage samples is higher in adults with asthma and correlates with asthma severity^34^. Interestingly, HLFs from patients with airway hyper-responsiveness demonstrate greater overall production of ECM constituents than those from healthy individuals^4,35^. Using a similar BEC/HLF co-culture model system, our group has previously reported that that HLFs co-cultured with BECs obtained from asthmatic donors drive co-cultured HLFs to produce greater amounts of important ECM constituents including types I and III collagen and HA^12^, and that HLFs co-cultured with BECs obtained from asthmatic donors displayed a greater degree of FMT compared to HLFs co-cultured with BECs obtained from healthy donors^13^. In recent studies we have provided both indirect and direct mechanistic evidence that airway epithelial TGFβ_2_, PGE_2_, and follistatin-like-3 (FSTL3)/activin A signaling influence FMT and HLF expression of ECM constituents^12,13,36^. In the present study, the positive association between PGE_2_ concentrations in co-culture supernatant and measures on lung function among asthmatic BEC donors provides additional evidence in support of the hypothesis that epithelial PGE_2_ may play a role in regulating FMT and HLF expression of ECM constituents, although future studies are needed to more directly test this potential mechanism by blocking epithelial PGE_2_ signaling. The novel observations of the current study are that we have for the first time demonstrated associations between *ex vivo* BEC-HLF crosstalk and clinical features among asthmatic BEC donors, namely lung function measures and history of severe asthma exacerbations.

Despite the unique strengths of our study design, it does have some important limitations. The subjects recruited into our cohort displayed mild airflow obstruction, and only 5 subjects exhibited bronchodilator responsiveness, indicative of a milder asthma phenotype. It is important to note that baseline spirometry obtained in our asthmatic subjects was significantly lower for all measured parameters compared to healthy subjects. Measurements obtained in our cohort may be indicative of an asthmatic population that has been appropriately treated with maintenance therapies as the majority of our subjects were taking daily inhaled corticosteroids. Given that only a small minority of asthmatic BEC donors exhibited bronchodilator responsiveness, we had insufficient power to assess potential associations between outcome measures in our BEC-HLF model system and bronchodilator responsiveness among BEC donors. Given that BECs were used in co-culture studies after two passages and several media changes, the likelihood that any residual corticosteroids would remain present in the culture media is extremely low; however, we are unable to fully exclude the possibility that lasting effects of these medications via epigenetic or other mechanisms exist in this model system. If these effects were present, they would likely bias data toward the null hypothesis. Studies designed to test the effects of steroids in BECs *ex vivo* will be an important future direction of investigation. For this study, we used a common HLF cell line from a healthy individual in all experiments to limit biological variability and isolate the differences to the primary BECs. We acknowledge that there are likely important differences in behavior in HLFs obtained from asthmatic and healthy children; however, this is beyond the scope of our present study. Finally, in this study, we provide indirect evidence to support a role of epithelial PGE_2_ as a regulator of airway FMT and HLF production of collagen I, however, we did not directly test this mechanism through blockade of epithelial-derived PGE_2_, which we hope will be an important aspect of future investigations using our model system.

## Conclusions

In this study, we have demonstrated an inverse correlation between expression of the myofibroblast marker α-SMA and COL1A1 by HLFs co-cultured with differentiated BECs obtained from asthmatic children and measures of lung function from BEC donors, and that HAS2 expression by HLFs co-cultured with BECs was greater when HLFs were co-cultured with BECs from asthmatic donors with a history of severe exacerbation. These novel findings relating *ex vivo* BEC-HLF cross-talk to clinical features among BEC donors, together with our previous reports of asthmatic BECs driving an activated, pro-remodeling phenotype in healthy HLFs^12,13^ in *ex vivo* studies, are consistent with airway biopsy data obtained from children with asthma, and provide additional translational data implicating the airway epithelium and airway remodeling as factors leading to lower lung function among children with asthma^5–8^. Additional studies are needed to further elucidate the signaling mediators involved in the intercellular crosstalk between BECs and HLFs (e.g., blockade of epithelial derived PGE_2_) and may provide valuable insight into novel therapeutic targets to prevent the establishment of airway remodeling in asthma.

## Methods

### Subjects

Children ages 6–18 years who were undergoing an elective surgical procedure requiring endotracheal intubation and general anesthesia were recruited for this study as either atopic asthmatic or healthy non-atopic non-asthmatic subjects. A detailed medical history was obtained to ensure that participants met the following inclusion/exclusion criteria. Children with asthma had at least a 1-year history of physician-diagnosed asthma, used a short-acting beta-agonist ≥twice a month or were taking a daily maintenance medication, and were not premature. Children with asthma had one or more of the following atopic features: history of a positive skin prick test or positive RAST for a common aeroallergen, elevated serum IgE (>100 IU/mL), history of physician-treated allergic rhinitis, history of physician-treated atopic dermatitis. Healthy subjects lacked a history of any of the above features and were excluded if they had any other history of atopy.

A blood sample was drawn from each subject and used to measure total serum IgE and RAST allergen-specific IgE to dust mites (*D. farina* and *D. pteronyssinus*), cat epithelium, dog epithelium, *Alternaria tenuis*, *Aspergillus fumigatus*, and timothy grass. At a subsequent follow-up visit, FE_NO_ was measured according to American Thoracic Society (ATS) guidelines using a NIOX MINO nitric oxide analyzer (Aerocrine®, Sweden)^37^. Spirometry was performed using a VMAX® series 2130 spirometer (VIASYS Healthcare, Hong Kong) to quantify FVC, FEV_1_, and FEF_25–75_ according to ATS guidelines. In children with asthma, spirometry was repeated 15 minutes following administration of 2 puffs of albuterol.

Written informed consent was obtained from a parent or legal guardian for all subjects below the age of 18 years, or from the subject if age 18 years. In addition, written assent was obtained for children ≥age 10 years. This study was approved by the Seattle Children’s Hospital Institutional Review Board.

### Establishment of BEC Cultures and BEC-Fibroblast Co-Cultures

Methods for BEC/HLF co-cultures have been described previously^12,13^. Briefly, BECs were obtained from donors following intubation for elective surgery. Cells were expanded in submerged culture and then passaged into transwells and differentiated at an ALI for 3 weeks. Co-cultures were established using commercially available pediatric HLFs (Lonza, Walkersville, MD) and were maintained for 96 hours at which point samples were isolated.

### RNA Extraction and quantitative PCR

Total RNA was isolated from HLF cells co-cultured with BECs grown at an ALI as previously described^12^. qPCR was performed using validated TaqMan® probes (Life Technologies, Grand Island, NY) for COL1A1, HAS2, and α-SMA using standard methodologies.

### Quantification of Collagen I, Hyaluronan, and PGE2 in Supernatant

Supernatant from BEC-HLF co-cultures was collected at the conclusion of experiments and frozen at −80 °C until constituents were measured. BEC-HLF supernatant concentrations of pro-collagen Iα1 were measured using an Enzyme-Linked Immunosorbent Assay (ELISA) from Abcam, Inc. (kit #ab210966, Cambridge, MA) per manufacturer instructions, concentrations of PGE_2_ were measured using an ELISA from Cayman® Chemicals (Ann Arbor, Michigan) per manufacturer instructions, and concentrations of hyaluronan (HA) were measured using an ELISA from R&D Systems® (kit DY3614-05; Minneapolis, MN).

### Flow cytometry

In a subset of BEC-HLF co-cultures (n = 10 asthmatic, n = 8 healthy), HLFs were mechanically detached from the culture wells and suspended in PBS. Samples were snap frozen in liquid nitrogen until use. Flow cytometry was used to quantify the percentage of myofibroblasts among HLFs as determined by cells staining positive for cytoskeletal α-SMA as we have previously described^13^.

### Immunohistochemistry (IHC)

Sterilized 12 mm round glass coverslips were coated with type I collagen and placed in the bottom of one of the replicate chambers of the 12-well plates prior to seeding the HLFs. Following 96 hrs. of co-culture, the coverslips were carefully removed and placed in a separate 12-well plate for IHC. Coverslips containing cells were then washed 3 times in room temperature PBS to remove residual media and were then fixed with 50:50 methanol and acetone at 20 °C for 10 minutes. Coverslips were then washed with PBS and blocked with 10% FBS for 30 minutes. Following this, coverslips were washed again with PBS and then incubated with an α-SMA-FITC primary antibody (clone 1A4, 1:500; Sigma Aldrich, St Louis, MO) as we have previously described^13^.

### Statistical Analysis

For RT-PCR studies, the relative expression of genes was normalized using GAPDH as a non-regulated reference gene. Analyses of RT-PCR results were performed using GenEx version 5.0.1 (MultiD Analyses AB, Göteborg, Sweden) based on methods described by Pfaffl^38^ and Prism® 6.0 software (GraphPad Software Inc., San Diego, CA). For comparisons between groups, unpaired t-test was used for normally distributed data, and the Mann-Whitney test was used for non-normally distributed data. To assess correlations between gene expression and lung function parameters, the Pearson correlation coefficient was used for normally distributed data, and the Spearman correlation coefficient was used for non-normally distributed data.

### Ethics approval and informed consent

Approval: The work presented in this study was approved by the Seattle Children’s Hospital Institutional Review Board. Accordance: The methods were carried out in accordance with relevant guidelines and regulations of the Seattle Children’s Research Institute and the Seattle Children’s Hospital Institutional Review Board. Informed consent: written informed consent was obtained from a parent or legal guardian for all subjects below the age of 18 years, or from the subject if age 18 years. In addition, written assent was obtained for children ≥age 10 years.

## Electronic supplementary material

Dataset 1

## Data Availability

The primary quantitative PCR and clinical lung function datasets used and/or analyzed for this study are provided in Dataset 1 of Electronic Supplementary Material.
