# Supplementary material for: Fibroblast gene expression following asthmatic bronchial epithelial cell conditioning correlates with epithelial donor lung function and exacerbation history
Source: Sci Rep. 2018 Oct 25;8:15768. doi: 10.1038/s41598-018-34021-6 (PMC6202408; doi:10.1038/s41598-018-34021-6)
Supplement: Supplementary file 1 — Dataset 1 [file 41598_2018_34021_MOESM1_ESM.pdf]

Title: Fibroblast gene expression following asthmatic bronchial epithelial cell conditioning correlates with epithelial donor lung function and exacerbation history

Authors: Stephen R. Reeves MD, PhD[1,2,3], Kaitlyn A. Barrow, BA[2], Tessa K. Kolstad, BS[2], Maria P. White, BA[2], Lucille M. Rich, BS[2], Thomas N. Wight, PhD[4], Jason S. Debley MD, MPH[1,2,3]

From the [1]Division of Pulmonary Medicine, Seattle Children's Hospital, Seattle, WA.; [2]Center for Immunity and Immunotherapies, Seattle Children's Research Institute, Seattle, WA.; [3]Department of Pediatrics, University of Washington, Seattle, WA; [4]Matrix Biology Program, Benaroya Research Institute, Seattle, WA.

Supplementary Information

	QPCR Ct values				alpha-SMA FMT by flow cytometry (% positive cells)	Collagen I ELISA (ng/mL)	HA ELISA (ng/mL)	PGE2 ELISA (pg/mL)	Lung function parameters (FEV1 % predicted, FEF25-75 % predicted, FEV1/FVC ratio)			
**BEC Donor**	**GAPDH**	**SMA**	**COL1A1**	**HAS2**					**FEV1**	**FEF25-75**	**FEV1/FVC**	
Healthy 1	18.21	24.45	21.46	26.60	2.79	2015.9	4391.0	2594.7	99	96	0.84	
Healthy 2	18.73	25.34	23.07	26.30		1478.8	8792.2	5917.9	139	104	0.91	
Healthy 3	18.60	24.91	22.23	27.17	3.54	771.0	3717.9	4503.4	115	106	0.88	
Healthy 4	18.96	24.63	21.85	26.96		1461.4	4755.5	4870.2	109	63	0.77	
Healthy 5	18.08	24.18	21.64	26.12		1383.9	8733.3		113	130	0.97	
Healthy 6	17.19	23.47	20.59	25.03	3.21	1863.5	6380.4	347.3	121	119	0.91	
Healthy 7	18.06	24.92	21.85	27.32		485.0	1200.9	3409.2	99	91	0.95	
Healthy 8	18.76	25.79	23.33	28.34	4.74	294.5	2024.9	5560.0	81	99	0.89	
Healthy 9	21.59	27.34	24.02	26.40					115	83	0.81	
Healthy 10	20.54	25.99	23.29	25.84					111	77	0.84	
Healthy 11	21.16	29.62	25.43	29.65					102	97	0.87	
Healthy 12	20.12	28.28	23.08	25.68	2.1				105	79	0.85	
Healthy 13	20.08	25.63	22.91	27.14	5.8	2867.8	3893.2	530.9	92	80	0.90	
Healthy 14	19.72	25.37	22.40	27.72	2	2567.8	5077.7	133.2	113	138	0.98	
Healthy 15	19.14	25.63	22.49	26.94	1.60	1665.7	3321.3	127.9	107	104	0.88	
												**History of Severe Exacerbation**
Asthma 1	20.07	25.06	22.60	25.62		1235.0	16978.0	1390.0	79	55	0.75	Yes
Asthma 2	19.97	24.78	22.47	25.91		1311.7	12590.9	4123.0	98	77	0.79	Yes
Asthma 3	20.92	25.75	21.81	26.37	9.31	1593.8	3166.0		82	59	0.82	Yes
Asthma 4	19.01	24.12	22.23	25.84	2.30	1138.3	7701.6	3960.5	85	82	0.85	No
Asthma 5	18.40	24.12	21.43	26.36		1682.5	10534.2	33.7	79	68	0.81	No
Asthma 6	19.57	25.20	21.64	26.97	5.86	936.4	6384.7	1911.1	94	74	0.91	Yes
Asthma 7	19.73	25.52	21.91	26.44	5.35	2381.2	14059.0	499.1	89	81	0.83	Yes
Asthma 8	18.92	24.99	21.48	27.17	3.20	910.4	4396.6	4653.2	116	105	0.90	Yes
Asthma 9	17.50	23.84	21.09	25.77	4.40	1690.0	10037.4	4024.6	113	98	0.85	No
Asthma 10	19.87	26.05	23.25	28.27		1473.6	6531.8	1247.3	92	78	0.83	No
Asthma 11	17.85	24.22	22.88	26.54	11.90	757.9	5554.6	3637.2	85	89	0.84	No
Asthma 12	20.29	25.43	22.06	25.54	10.30	6466.9	2842.6	1151.6	58	34	0.64	Yes
Asthma 13	20.50	25.35	22.11	24.80	8.10	2120.5	3217.9	1551.0	82	61	0.75	Yes
Asthma 14	20.22	25.33	22.50	24.05					78	101	0.84	Yes
Asthma 15	20.76	25.99	22.90	26.04					89	82	0.75	Yes
Asthma 16	21.26	25.96	20.34	28.07	14.50	3994.4	3932.8	614.0	77	47	0.79	No
Asthma 17	20.63	25.30	20.01	27.20		4152.5	5797.5	143.6	86	66	0.79	No
Asthma 18	20.94	25.80	19.92	27.75		5191.9	3809.1	35.3	64	58	0.79	Yes
Asthma 19	20.55	25.23	19.71	27.11		11141.1	5114.4	502.2	73	42	0.7	No
Asthma 20	20.69	25.41	19.87	27.27		4154.6	5262.7	805.9	88	63	0.76	No
Asthma 21	20.94	25.06	20.14	27.83		2842.1	3739.9	568.8	78	59	0.73	No
Asthma 22	21.11	25.81	20.15	27.59		4765.2	2737.3	1189.3	85	78	0.77	Yes
Asthma 23	20.56	25.68	20.49	26.97		3380.2	4072.0	2079.4	97	70	0.7	Yes
